# Rehabilitation of Gait and Balance in Cerebral Palsy: A Scoping Review on the Use of Robotics with Biomechanical Implications

**DOI:** 10.3390/jcm12093278

**Published:** 2023-05-04

**Authors:** Mirjam Bonanno, Angela Militi, Francesca La Fauci Belponer, Rosaria De Luca, Danilo Leonetti, Angelo Quartarone, Irene Ciancarelli, Giovanni Morone, Rocco Salvatore Calabrò

**Affiliations:** 1 IRCCS Centro Neurolesi “Bonino-Pulejo”, Via Palermo, SS 113, C. da Casazza, 98124 Messina, Italy; 2 Department of Biomedical and Dental Sciences and Morphological and Functional Imaging, University of Messina, 98125 Messina, Italy; 3 Neuropsichiatria Infantile, Azienda Ospedaliera Universitaria (AOU), Policlinico “Gaetano Martino”, 98125 Messina, Italy; 4 Department of Biomedical, Dental and Morphological and Functional Images, Section of Orthopaedic and Traumatology, University of Messina, 98125 Messina, Italy; 5 Department of Life, Health and Environmental Sciences, University of L’Aquila, 67100 L’Aquila, Italy; 6 ASL 1 Abruzzo (Avezzano-Sulmona-L’Aquila), 67100 L’Aquila, Italy; 7 San Raffaele Institute of Sulmona, 67039 Sulmona, Italy

**Keywords:** cerebral palsy, robotic neurorehabilitation, balance and gait disorders, biomechanical gait parameters, gross motor functions

## Abstract

Cerebral palsy (CP) is a congenital and permanent neurological disorder due to non-progressive brain damage that affects gross motor functions, such as balance, trunk control and gait. CP gross motor impairments yield more challenging right foot placement during gait phases, as well as the correct direction of the whole-body center of mass with a stability reduction and an increase in falling and tripping. For these reasons, robotic devices, thanks to their biomechanical features, can adapt easily to CP children, allowing better motor recovery and enjoyment. In fact, physiotherapists should consider each pathological gait feature to provide the patient with the best possible rehabilitation strategy and reduce extra energy efforts and the risk of falling in children affected by CP.

## 1. Introduction

Cerebral palsy (CP) is a congenital and permanent neurological disorder due to non-progressive brain damage [1] that may occur from brain malformations, preterm white matter injury, hypoxic-ischemic injury, pre-, peri-, and postnatal stroke, CNS infection, or traumatic brain injury [2]. CP are leading cause of various motor impairments, including a mixture of positive (increased muscle tone) and negative (insufficient active muscle control) neurological signs that worsen children’s quality of life (QoL) [3]. Balance, trunk control, and gait alterations are the most affected gross motor functions (GMFs) that reduce independence in CP patients [4]. Specifically, children with CP may present some biomechanical alterations during gait, which depend on reduced knee flexion in swing phase of gait, knee hyperextension (i.e., recurvatum) or excessive flexion (i.e., crouch) during the stance phase of gait, in conjunction with limited dorsiflexion (i.e., equinism), leg crossing in swing (i.e., scissoring), internal hip rotation, and excessive hip adduction [5]. All these abnormalities, in addition to balance disorders and reduced trunk control, may alter foot placement and the correct direction of the whole-body center of mass (CoM) with stability reduction and an increase in falling [6,7]. For these reasons, neurorehabilitation has been developed and improved to meet the needs of each patient, although nowadays, there is no standardized protocol for treating altered GMFs, such as gait and balance in CP patients [8].

Conventional neuromotor techniques, including Bobath and Vojita [9], as well as medical treatments for spasticity (i.e., botulinum toxin) [10], have been developed to improve muscle strengthening, increase joint range, reduce stiffness and spasticity, and promote coordination and balance. Additional standard treatments for CP patients include strength training, which increases gait speed, lower limb muscle strength and balance without affecting spasticity [11]. However, these conventional approaches have some limits, such as the lack of a standardized training environment, the possibility to adequately increase therapy intensity and dose, as well as the short-term duration of their effects and high staff costs and efforts. In fact, conventional physiotherapy sessions can be more challenging, especially for the repeatability of the exercises and the spatial and temporal symmetry of steps. Moreover, the evaluation of biomechanical gait and balance parameters often require specific technology. To overcome these concerns, robotic systems have been employed as emerging rehabilitative tools that can provide an early rehabilitation program [12] with high-intensity, repetitive, task-specific and interactive training. It is noteworthy that rehabilitation robots have been used as an effective therapy to improve GMFs, such as trunk control, balance and gait in patients affected by CP [13].

The aim of this scoping review is to investigate the existing literature about the effects of robotic systems in improving gait and balance functions in patients with CP, focusing on the biomechanical implications for physiotherapy clinical practice.

## 2. Materials and Methods

**Figure 1:**
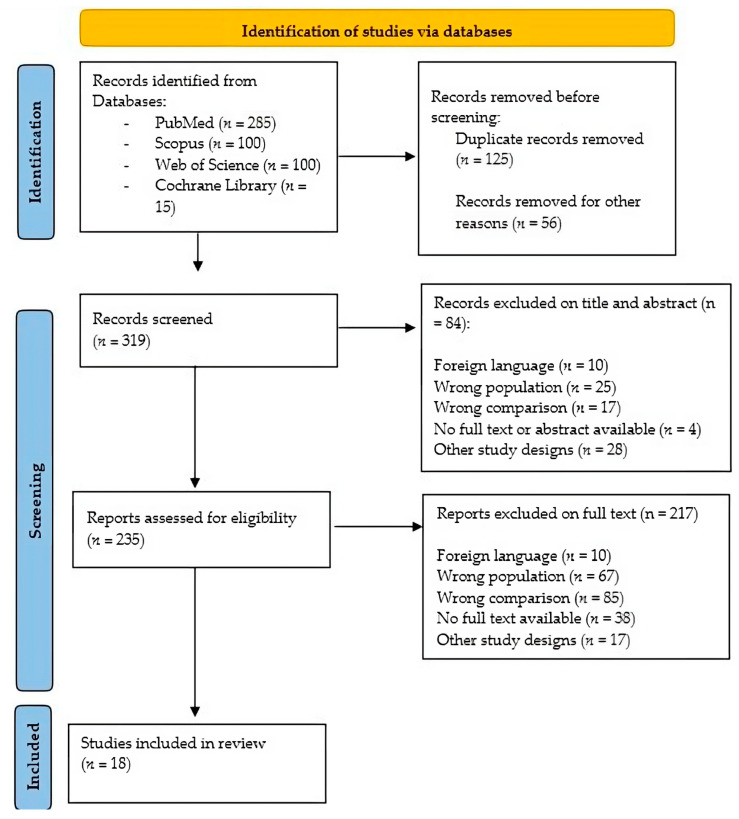
PRISMA flow diagram for study selection.

According to the acronym PCC (Population/Problem, Concept, Context), our research questions were the following “How robotics improves balance and gait in Cerebral palsy, and what are the biomechanical effects of robotic training in this population?”. We considered children and young adolescents affected by CP with gait and balance alterations as the Population/problem; the Concept was the application of robotic devices to train balance and gait, considering the biomechanical parameters; the context was the neurorehabilitation setting. The studies included in this review were identified by searching on PubMed, Scopus, Google Scholar and Cochrane Library (n = 500) using the following keywords: “robotic gait rehabilitation” AND “cerebral palsy” AND “robotic balance rehabilitation” AND “cerebral palsy” AND “robotic neurorehabilitation” OR “gross motor function neurorehabilitation”. We have included only English articles, and we analyzed the references to obtain a complete search. We selected articles between 2012 and 2022 and evaluated the papers according to title, abstracts and text (n = 319), removing duplicates (n = 125). Finally, we considered 18 articles that dealt with robotic gait and balance neurorehabilitation in the CP population, also considering the biomechanical issue, as we reported in Figure 1 according to new PRISMA statements [14] (Figure 1).

In detail, two reviewers (MB and AM) involved in the research of the articles carried out their activity blind to each other, according to the following inclusion criteria: (1) children and adolescents patients affected by CP; (2) Robotic training sessions for balance and gait; (3) Evidence that measured biomechanical parameters during a training session. Exclusion criteria were (1) children and adolescents with other neurological disorders; (2) patients affected by psychiatric alterations and comorbidities; (3) Not-specific evidence about biomechanical issues and/or balance and gait. After that, the two reviewers read the selected articles individually and confirmed or rejected their inclusion in the review; when there was a doubt about inclusion, a third reviewer (RSC) was involved in solving the concern.

## 3. Results

We analyzed the 18 selected articles that met our inclusion criteria. We divided the results into two main sections based on specific GMF recovery: nine studies dealt with robotic balance training, and nine studies involved robotic-assisted gait training (RAGT) (Table 1).

### 3.1. Trunk Control and Balance Robotic Training

When the injury affects the brain areas involved in the segmental control of the trunk, children with CP could present difficulties in achieving unsupported sitting due to control deficits of low thoracic and lumbar regions that disrupt posture and balance [15]. In this context, a few patients could benefit from external support, such as robotic devices, targeting impaired postural control. Santamaria et al. [16] delivered a postural task-oriented training through a motorized cable-driven belt, called Trunk-Support-Trainer (TruST), placed on the CP child’s most-impaired trunk region. The TruST provides maximum trunk displacement in the reaching task **to** promote postural reactions. In fact, it can force the patient to develop active trunk movements. This training produced clinical improvements in GMFs, as well as in postural and reaching control, allowing children to sit independently. Abidin et al. [17] studied the effects of a combined approach using RAGT and standard physiotherapy. The authors found that RAGT, as an adjunct treatment to physiotherapy, was useful in promoting trunk control, sitting balance and posture in non-ambulatory CP. RAGT using the Lokomat device has proven effective in enhancing cortical plasticity and cerebellar-motor connectivity through augmented sensory and proprioceptive feedback [18]. In line with this hypothesis, it has been confirmed [19] that training with the electromedical device Alter-G can favor plastic changes in the brainstem, cerebellar white matter and vestibulospinal tract, producing permanent postural and balance improvement in CP children [20]. The mechanical horse-riding simulator (HRS), an emerging type of intervention based on hippotherapy and consisting of a robotic device with a dynamic saddle that imitates the movement of a horse [21], is of particular interest in this field. According to a systematic review [22], HRS seems to be effective in improving balance in subjects affected by CP, especially in terms of anteroposterior, medial–lateral weight shifting, trunk decompensation, inclination and pelvic torsion. In fact, the riding movement produces soft, rhythmic, and repetitive patterns, simulating the pelvis movements during normal human walking [23,24,25]. These kinds of repetitive riding movements could improve postural coordination through the stimulation of balance reactions thanks to the effort to maintain the center of gravity inside the support base [25,26]. In a single case study carried out on a child with CP [27], HRS was also effective in increasing postural muscle tropism, including internal oblique, external oblique and lumbar multifidus, with static and dynamic stability. Indeed, the use of HRS could be a valid device to manage hip adductor spasticity, which worsens motor functions and postural control, as confirmed by Hemachithra et al. [28]. The authors showed that HRS was effective in decreasing the adductor spasticity and improving the abduction range of motion in the hip, suggesting its use within physiotherapy intervention. Recently, Jung et al. [29] investigated the use of HRS combined with virtual reality (VR) in a sample of preschool- and school-aged children with spastic CP. The authors found that the combined approach was more effective in improving GMFs, balance control, and mobility without serious adverse events, rather than only HRS training.

### 3.2. Robotic Gait Training: A Biomechanical Perspective

**Figure 2:**
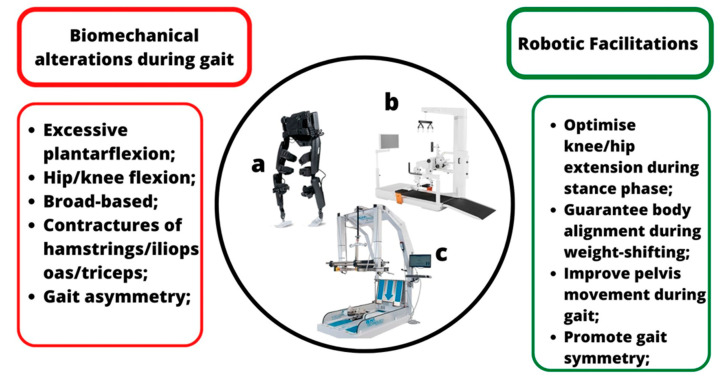
Description of biomechanical alterations during gait in CP and possible solutions provided by robotic devices: (**a**). wearable Exoskeleton (i.e., Ekso); (**b**). Exoskeleton with a treadmill (i.e., Lokomat); (**c**). Robotic end-effector (GE-O System).

In patients with CP, gait is often accompanied by reduced pendular synkinesis of the upper limbs, stiffness and imbalance, which require more energy and effort rather than **in** healthy children [30]. The development of RAGT attempts to achieve a correct motor function since robotic tools can provide high-intensity, repetitive, task-specific, and interactive training. Generally, two types of robotic gait devices can be distinguished in clinical practice: end-effectors and exoskeletons. End-effectors are stationary devices that reproduce gait trajectories through footplates guiding feet (i.e., GE-O System) and the swinging phase of gait. Exoskeletons are wearable devices for both over ground (i.e., Ekso) and on a treadmill (i.e., Lokomat) walking. The main difference between these two robotic gait devices is that end-effectors only act on the distal part of the body (i.e., feet), while exoskeletons act on the main joints of the lower limb (Figure 2) [31].

With recent advances in technology, RAGT has become more readily accessible and available in rehabilitation centers. Evidence suggests trends for improvement of muscle activation after active training performed with high intensity and rate of guided movements [32]. According to Wallard et al. [33], it seems that RAGT could promote new dynamic strategies of gait, which are characterized by more appropriate control of the upper body in addition to improvement of the lower limb kinematics. However, it has been pointed out [34] that RAGT is no more effective than standard physiotherapy, especially in the case of passive gait training without the active engagement of the patient necessary to promote motor learning. This is why active walking training (above all when it is used robotics plus VR) could enhance cortical activity and plasticity due to higher attention required with a better modulation of gait speed and steps, as confirmed by Yazıcı et al. [35]. The degree of gait impairment is another factor that cannot be underestimated. In fact, it has been found [36] that RAGT, using the Robogait (Bama Technology, Ankara, Turkey), promoted improvements in the standing and walking abilities only in mild to moderate CP children. Notably, wearable robots facilitate an appropriate alignment of the body axis during weight-shifting movement, improving both static and dynamic balance control and, therefore, gait function. Indeed, it seems that exoskeletons could be more effective for dynamic balance than tethered-type robots [37]. A recent systematic review [38] supported their use to optimize the knee and hip extension during the stance phase, reducing the metabolic cost of gait and increasing knee flexor and extensor muscle activity in children with CP. It seems that wearable exoskeleton devices could facilitate the activation of trunk muscles to guarantee an appropriate body alignment during weight-shifting movement, improving both static and dynamic balance, as well as gait [39]. In this vein, Kawasaki S. et al. [40] revealed that RAGT using an exoskeleton increased hip joint angles on the affected limb side promoting gait symmetry. In this way, patients were able to produce a better-grounded propulsion force, which is strictly correlated with plantar flexor strength. Interestingly, some authors [41] found that even a low dose of RAGT, using the Lokomat, could improve hip flexors and knee extensors muscle strength in moderate to severe CP patients.

## 4. Discussion

**Figure 3:**
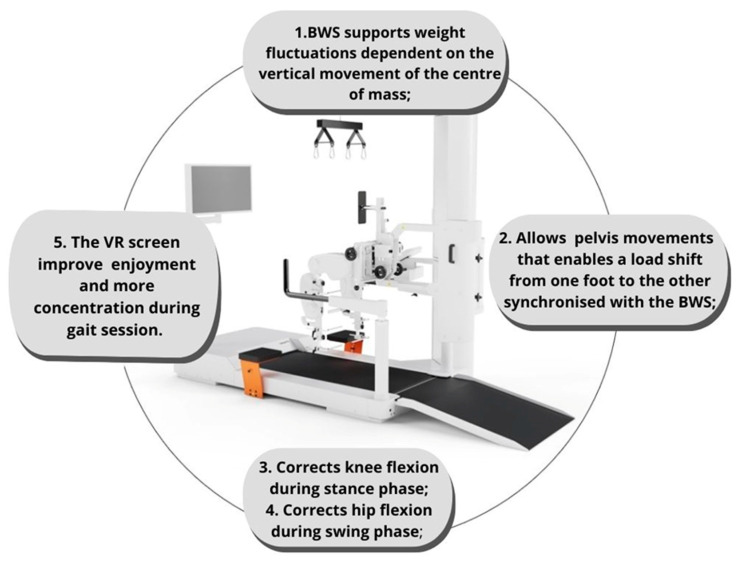
Illustrates biomechanical facilitations for CP patients using Lokomat.

Balance and gait impairments are the most common motor deficits in patients affected by CP. Several robotic devices can facilitate the recovery of these fundamental motor functions, meeting the biomechanical features of each “frail” CP child. Indeed, most existing studies focused on gait training, while postural and balance recovery remained overlooked. Noteworthy, although the Lokomat (Figure 3) device is the most used RAGT device [42], no study was found that compared its superiority with other robotic devices.

Lokomat seems to induce improvements also in balance outcome measures since gait and balance are strictly correlated due to the nature of bipedal locomotion. However, these improvements in balance through the device can be linked to the idea of the “reverse transfer”. This latter proposes that repeated practice of high-intensity walking training may improve non-walking tasks, such as static balance and postural stability [18,43]. Indeed, the Lokomat device has the advantage of promoting CP children’s motivation and attention through virtual reality (VR) screens. VR settings are pleasant, educational and safe and can improve concentration, as confirmed by Cho et al. [44]. Then, the presence of VR helps to reduce the patient’s effort by increasing the muscle strength of lower limbs and gait velocity [44], thanks to the enjoyment and involvement during the training, which allows longer rehabilitation sessions, as compared to standard RAGT alone [45,46]. In fact, it has been shown that CP patients trained with VR have gained better control of the right foot placement based on the loading of body weight [7].

Nonetheless, RAGT can improve gait symmetry more than spatiotemporal gait parameters and postural responses. In fact, to improve postural and balance functions, more signals from the proprioceptive and vestibular receptors are needed. In this vein, the adjunct of VR to robotics [29,44] becomes more challenging for CP children who are forced to activate lower limb and hip muscles, as well as trunk muscles, compensating for the continuous postural changes due to moving the saddle.

Although robotic devices are common in hospital settings, they require consistent investments in terms of maintenance and routine operation [47]. This is why not all hospitals can afford the expense of their implementation, representing a barrier, especially in developing countries. Volpini et al. [48] developed a simple, low-cost prototype of a robotic device (i.e., robotic orthosis equipped with the treadmill) for the gait training of children with CP. This prototype allows registering spatiotemporal parameters of gait and comparing them with those of healthy children. Notably, the device presents two systems to guide ankle movements during the kinematic trajectory of gait. Then, the authors [48] found a less expensive strategy to train children of 7–10 years old affected by CP, with robotics ensuring intensity, repetitive and long gait sessions, which are fundamental to potentiate brain plasticity.

Other non-invasive technologies used to influence cortical activity in CP children consist of brain stimulation through transcranial direct current stimulation (tDCS) and repetitive transcranial magnetic stimulation (rTMS). According to a systematic review [49], tDCS seems to be particularly effective in modulating cortical excitability to promote balance and gait recovery (especially gait velocity and cadence) in brain-injured children in addition to conventional physiotherapy. Coupling neuromodulation with robotics would help further increase functional outcomes in neurological patients, including CP.

In physiotherapy practice, the biomechanical issue should be considered to address the best rehabilitation strategy, especially in CP patients who present a high risk of tripping and falling during gait [50,51,52]. Gait function can be considered as the forward displacement of the body requiring coordination between alternate successions of the swing and the stance phases [33]. Robotic and/or VR devices allow therapists to evaluate some important measures of gait, including cadence, step and stride length, stride width and gait symmetry (see Table 1) and to adapt them to the patient’s necessities. Recently Ma et al. [53] evaluated gait features using a virtual environment (CAREN) in CP children as compared to healthy controls. They found that CP children tend to produce extra plantar flexion during the early stance phase (Table 1). For this reason, the authors suggested being careful in increasing speed during treadmill uphill walking, considering the joint effort for patients. To overcome these concerns, biomechanical adjustments are needed, considering the different patterns of gait alterations (Supplementary Table S1). Indeed, knowing how the biochemical parameters change in CP patients is fundamental to tailoring their rehabilitation, especially when innovative technologies are applied.

In fact, during RAGT, patients could necessitate a reduced walking speed and stride length, increased ankle dorsiflexion, hip flexion, and knee flexion (during the stance phase) [53,54,55] to improve gait and balance functions. The trunk plays a crucial role in postural control mechanisms and in balance reactions. In fact, trunk control is fundamental for the stride width or base of support, which is necessary during gait [56]. To this aim, robotics could meet biomechanical issues and patients’ needs, evaluating the musculoskeletal alterations and adapting training to them.

Eventually, there is no standardized protocol to train gait and balance based on the age of CP children and the level of motor function severity. This is why it is not easy to choose the right robotic device in clinical practice [57].

Herein, we reported some advice about the use of robotics according to the literature and based on the Gross Motor Function Classification System (GMFCS):

1. CP patients with levels I and/or II of GMFCS are characterized by fatigue over long walking distances with mild limitations in gait and balance. They, therefore, could benefit from a treadmill (with or without BWS) and/or Alter G for gait training to improve endurance and gait velocity. VR exercises may further stimulate motivation and longer rehabilitation sessions. In fact, virtual gaming platforms could enhance postural stability, also in patients with ataxic CP.
2. In level III, CP patients need more support to walk, especially outside. In this context, wearable exoskeletons, such as the Ekso-GT (Figure 2a), are useful to promote overground walking and autonomy in ambulation (when possible). The motor function can also be improved using the Lokomat, one of the most adaptable robotic tools in neurorehabilitation, thanks to its characteristics (Figure 3) that allow training patients with moderate to severe motor alterations. In addition, Lokomat equipped with a VR screen can provide more challenging training sessions, thus further increasing concentration but also enjoinment.
3. CP children between III and IV GMFCS levels present more balance and gait limitations, and the active achievement of an upright position is not always possible. This is why they could benefit from gait training with Innowalk Pro, which supports the patients from sitting to verticalization, in addition to a BWS system. Moreover, exoskeletons like the Lokomat and/or Robogait may help in guaranteeing gait movements in a more passive modality, also reducing efforts for little patients.
4. CP patients with level IV present important motor impairments, which tend to confine them to wheelchairs. Then, they could benefit from passive robotic gait training using exoskeletons equipped with BWS (i.e., Lokomat and Robogait), providing constant assistance and monitoring vital signs (blood pressure and oxygen saturation). In addition, the use of HRS with or without VR can be a valid instrument to improve balance functions, postural reactions and abduction hip ROM.

## 5. Conclusions

Robotic devices to train GMFs, such as balance and gait, are various and seem to be effective in improving CP outcomes, in addition to conventional physiotherapy. To date, no specific recommendation exists on which kind of device is better, as well as on the intensity, duration and protocol of gait training in these frail patients. Nonetheless, children more severely affected may benefit from exoskeletons (since they have better joint and trunk control), whereas less impaired CP children may be trained with end-effectors and VR devices (as they require spared motor function). In this context, biomechanical issues are fundamental to carrying out the best possible therapeutic strategy, personalizing each training session, and improving patient outcomes and quality of life.

## Figures and Tables

**Table 1:** Description of selected studies based on the type of robotic device and clinical/biomechanical evaluation.

Reference n°	Robotic Device	Clinical Evaluation	Biomechanical Parameters
Robotic balance training			
16	TruST—cable-driven robot which provides assist-as-needed forces.	SP&R-co; BBT; MFRT;	Lower-thorax orientation during sitting (flexion–extension plane)
17	RAGT (authors not specified the robotic system)	GMFM and TIS	Sitting balance
18	Lokomat—(description focused on balance) a fixed gait rehabilitation exoskeleton which facilitates a bilateral symmetrical gait, as the individual actively tries to advance each limb, during walking.	BBS, TUG, RMI, mEFAP, MM, PASS, POMA-B, SPPB, RMA	Walking speed and covered distance.
19	AlterG—it is a treadmill which enables patients to walk/run at low percentage of their body weight, thanks to its microgravity envinronment.	Romberg test and DTI evaluation	Center of Pressure (CoP)
20	AlterG—mentioned above	BBT, TUG, DTI evaluation	Balance and posture
22	HRS—is a robotic device with a dynamic saddle.	Stabilometric platform, GMFM, Sitting assessment scale (SAS), BBS, B-POMA, SATCo, Romberg test	Balance, pelvic torsion, and pelvic tilt
23	Horseback riding simulator JOBA–robotic simulator of horse movement.	NA	Spinal posture: trunk imbalance, pelvic torsion, and pelvic tilt
27	Robotic HPOT system (FORTIS-102)—This robotic system simulates live horse movements (I.e. walk, trot, and gallop) and the partecipant is instructed to the different patterns of movement.	Ultrasound imaging of EO, IO, TrA, LM.	CoP, CoM
28	horse riding simulator U-Gallop—indoor exercise equipment, which uses the oscillatory action of the seat to simulate the horse riding experience.	MAS and PROM in hip abduction	NA
Robotic assisted gait training evidence			
13	Lokomat—(description focused on gait) robotic exoskeleton with active hip–knee actuation and passive ankle control during the swing phase, in addition to a variable level of assistance.	GMFM and Cerebral Palsy Quality of Life Questionnaire (CP QOL)	NA
33	Lokomat—mentioned above	Clinical gait analysis and GMFM	COP, COM, propulsive forces in anteroposterior and Medio lateral directions
34	RAGT (authors not specified the robotic devices)	GMFM, 6MWT	Walking speed,
35	Innowalk Pro—it is an end-effector that supports the user from sitting into a standing position, and provides assisted, guided and repetitive movements in a safe upright, weight-bearing position.	GMFM, 10MWT, 6MWT, standing on one leg test, PBBS, FAQ-WL	Walking speed and endurance, balance,
36	Robogait— is a fixed lower body hip-knee exoskeleton. The user’s weight is supported by a combination of an overhead attached harness and the support from the exoskeleton.	GMFM and 6MWT	Walking covered distance and gait velocity
38	tethered knee exoskeleton, pediatric knee exoskeleton (P.REX), untethered ankle exoskeleton, WAKE-Up ankle module, WAKE-Up ankle & knee module and unilateral ankle exosuit— It is not a passive robot but the device can interact actively with patients, correcting also the motor actions on the joint motion; in this way the subject is assisted or less only when requested.	EMG	Walking cadence, stride length and/or step length, gait symmetry, stride to stride vaiability
39	wearable joint-torque-assisting exoskeletal robot called the Angel Legs M20—wearable walking device that induce proper gait and support of the lower limbs.	GMFM, 6MWT,10MWT, Oxygen consumption	Walking speed
40	HWA (exoskeleton that assists hip flexion and extension of both limbs during gait)—it detects the gait cycle by the potentiometers (set beside the actuators) and produces flexion and extension torques on swing and stance phases, respectively.	MAS, ROM and MVC of hip flexion, hip extension, knee flexion, knee extension, dorsiflexion, and plantar flexion, GMFM (D and E dimensions), PEDI	Walking speed and gait symmetry
41	Lokomat—mentioned above.	hand-held dynamometer to test strength of lower limb muscles	Walking speed, cadence, stride length and sagittal joint kinematic gait parameters: pelvis, hip, knee and ankle minimum and maximum angles, as well as range of motion (ROM) during the stance phase.

Legend: TruST (Trunk-Support-Trainer), SP&R-co (Seated Postural & Reaching Control Test), BBT(Box and Block Test), MFRT (Modified Functional Reach Test), BBS (Berg Balance Scale), TUG (Timed Up and Go), RMI (Rivermead Mobility Index), mEFAP (Modified Emory Functional Ambulation profile), PASS (Postural Assessment Scale for Stroke), MM (Mobility Milestones), POMA-B (Tinetti Performance-Oriented Mobility Assessment), SPPB (Short Physical Performance Battery), RMA (Rivermead Motor Assessment), DTI(Diffusion Tension Imaging), BBT(Berg Balance test), GMFM (Gross Motor Function Measure), SAS (Sitting assessment scale), SATCo (Segmental Assessment of Trunk Control), EO (external oblique), IO (internal oblique), TrA (transversus Abdominis, LM (lumbar multifidus), CoP (Centre of Pressure), CoM(Centre of Mass), MAS (Modified Ashoworth scale), PROM (Passive Range of Motion), CP QOL (Cerebral Palsy Quality of Life Questionnaire), 6MWT (Six Minute walking test), 10MWT (Ten Minute Walking Test), PBBS (Pediatric Berg Balance Scale), FAQ-WL (Gillette Functional Assessment Questionnaire Walking Scale), EMG (Electromyography), MVC (Maximum Voluntary isometric Contraction), PEDI (Pediatric Evaluation of disability Inventory).

## Data Availability

Not Applicable.

## Supplementary Materials

The following supporting information can be downloaded at: https://www.mdpi.com/article/10.3390/jcm12093278/s1, Table S1: Description of the main biomechanical gait parameters and the most common characteristics in CP during gait.
