# Supplementary material for: Rehabilitation of Gait and Balance in Cerebral Palsy: A Scoping Review on the Use of Robotics with Biomechanical Implications
Source: J Clin Med. 2023 May 4;12(9):3278. doi: 10.3390/jcm12093278 (PMC10179520; doi:10.3390/jcm12093278)
Supplement: Supplementary file 1 [file jcm-12-03278-s001.zip › jcm-2229558-supplementary.pdf]

Supplementary Table S1. Description of the main biomechanical gait parameters and the most common characteristics in CP during gait.

Biomechanical Gait Parameters	Description of Gait Characteristics in CP
**Cadence**	Cadence measures speed as the number of full gait cycles (on both right and left foot strike) during a given period of time and expressed as steps (or cycles) per minute.<br>In CP patients, cadence of gait is reduced by spasticity which affects the entire lower limb, increasing energy expense and fatigue reducing gait speed.
**Step length**	Step length is the distance between the point of initial contact of one foot and the point of initial contact of the opposite foot. In normal gait, right and left step lengths are similar.<br>Step length is altered due to the excessive plantar flexion of the ankle, which determines knee and hip flexion, or knee hyperextension, but also it can be caused by weakness/spasticity of the more proximal muscles of the lower limb.
**Stride length**	Stride length is the distance between successive points of initial contact of the same foot.<br>The stride length may be reduced due to excessive plantar or dorsiflexion of the ankle, or due to spasticity/contracture of the adductor muscle chain.
**Stride width**	Stride width, also called base of support, is the distance between the heels of the two feet during double stance. Stride width is measured either between the medial-most borders of the two heels or between lines through the midline of the two heels. Normal stride width for adults is between 1–3 inches (3–8 cm).<br>Stride width or base of support is particularly reduced due to decreased strength in the gluteus medius and adductors contracture leading to a 'scissor' gait associated with reduced postural stability and a high risk of femoral head dislocation.
**Stance phase**	The stance phase occupies 60% of the total gait cycle, during which some part of the foot is in contact with the ground. It is further divided into five sub-phases:<br>• Initial contact (heel strike);<br>• Loading response (foot flat);<br>• Mid-stance;<br>• Terminal stance (heel off);<br>• Pre-swing (toe off);<br>Excessive plantar flexion leads to knee, hip and trunk compensations in the stance phase, causing antepulsion of the trunk and high risk of falls.<br>In the crouch gait, excessive dorsiflexion of the ankle results in knee flexion during the stance phase, causing greater postural instability and increased fatigue.<br>[50–52]

**Swing phase**	The swing phase occupies 40% of the total gait cycle, during which the foot is not in contact with the ground and the body weight is borne by the other leg and foot. It is further divided into three sub-phases:<br>• Initial swing<br>• Mid-swing<br>• Late swing<br><br>In the swing phase, a toe drop can occur, in adjunct to an excessive hip flexion due to compensation.<br><br>While inadequate hip flexion during the swing phase can produce a number of compensations including: pelvic retroversion, circumduction of the lower limb and increased plantar flexion [48–50].
**Gait symmetry (GS)**	GS is characterised by the almost identical behaviour of bilateral limbs during a gait cycle. It is important in measuring gait pattern alterations for establishing the level of functional limitation due to pathology.<br><br>Gait in CP patients is highly asymmetrical due to musculoskeletal changes caused by spasticity and muscle contractures (i.e., hamstrings, triceps, iliopsoas).
